# Supplementary material for: High prevalence and risk factors of undernutrition in adult cancer patients at Hawassa University Hospital: a call for targeted interventions
Source: PeerJ. 2025 Sep 4;13:e19925. doi: 10.7717/peerj.19925 (PMC12422259; doi:10.7717/peerj.19925)
Supplement: Supplemental Information 2 [file peerj-13-19925-s002.docx]

**Code book**

- Each value represents an answer on the survey

| No | Question | Possible out put |  |
| --- | --- | --- | --- |
|  | NewAge3 | 1=>65, 2 <65 |  |
|  | Sex | 1=male,2=female |  |
|  | MaritalStatus | 1=single,2=married,3=divorced,4=widowed |  |
|  | Occupation1 | 1=farmer,2=merchant,3=government employe4=NGO,5=0ther |  |
|  | smoking | 1=yes,2=no |  |
|  | Family history of CA | 1=yes,2=no |  |
|  | comorbidity | 1=yes,2=no |  |
|  | typesofcancer | 1= Breast ca ,2=Gastro intestinal truct ca , 3=Sarcoma, 4=Lmphoma ,5=Gynycologic ca, 6=Lung ca, 7=HENT, 8=Hepatocellular ca. 9=Others |  |
|  | stage of cancer | 1=StageI, 2=StageII 3=StageIII 4=StageIV |  |
|  | Route of feeding1 | 1=Solid diet,2=semi solid diet |  |
|  | Wt of the participant | scale |  |
|  | Ht of partcipant | scale |  |
|  | cereals | 0=no,1=yes |  |
|  | WTR (White tuber and roots) | 0=no,1=yes |  |
|  | Vegetables | 0=no,1=yes |  |
|  | Fruits | 0=no,1=yes |  |
|  | Meat | 0=no,1=yes |  |
|  | Eggs | 0=no,1=yes |  |
|  | Fish | 0=no,1=yes |  |
|  | Legumes | 0=no,1=yes |  |
|  | MMP(Milk and milk product) | 0=no,1=yes |  |
|  | OaF (oil and fat) | 0=no,1=yes |  |
|  | sweets | 0=no,1=yes |  |
|  | CT (Cofee and tea) | 0=no,1=yes |  |
|  | MSH (meal or snack out of home) | 0=no,1=yes |  |
|  | ZPGSGA_N | 0=notundernouirished,1=undernourished |  |
|  | ZHgb_N | 0=not anemic,1=anemic |  |
|  | ZBMI_N |  |  |
|  | ZDDS_N | 0=High DDS,1=Low DDS |  |
|  | ZTfeeding_N | 0=Solid diet,1=Semi solid/liquid diet |  |
|  | ZAlcho_N | 0=no history of alcohol intake,1=have history of alcohol intake |  |
|  | Zappetite_N | 0=no loss of appetite,1=have loss of appetite |  |
|  | Znausea_N | 0=no nausea,1=have nausea |  |
|  | Zdysphagia_N | 0=no difficulty of swallowing,1=have difficulty of swallowing |  |
|  | Zedustat_N | 0=College and above,1=Secondary,3=primary,4=No formal education |  |
|  | Zkhat_N | 0=no history of khat chewing ,1=have history of khat chewing |  |
|  | Zsubabuse_N | 0=no history of substance abuse ,1=have history of substance abuse |  |
|  | Zconstipation_N | 0=no history of constipation ,1=have history of constipation |  |
|  | Zdiarhoea_N | 0=no history of diarrhea ,1=have history of diarrhea |  |
